# Clinicopathologic analysis of 14 cases of odontogenic myxoma and review of the literature

**DOI:** 10.4317/jced.52953

**Published:** 2017-04-01

**Authors:** Ana-Lucia-Noronha Francisco, Thiago-Celestino Chulam, Fábio-Oliveira Silva, Diogo-Gonçalves Ribeiro, Clóvis-Antônio-Lopes Pinto, Rogério-Oliveira Gondak, Luiz-Paulo Kowalski, João Gonçalves-Filho

**Affiliations:** 1 DDS, PhD, Department of Head and Neck Surgery and Otorhinolaryngology, A.C. Camargo Cancer Center, São Paulo, Brazil; 2 MD, PhD, Department of Head and Neck Surgery and Otorhinolaryngology, A.C. Camargo Cancer Center, São Paulo, Brazil; 3 MD, Department of Head and Neck Surgery and Otorhinolaryngology, A.C. Camargo Cancer Center, São Paulo, Brazil; 4 MD, PhD, Department of Pathology, A.C. Camargo Cancer Center, São Paulo, Brazil; 5 DDS, PhD, Department of Pathology, Federal University of Santa Catarina, Florianópolis, Brazil

## Abstract

**Background:**

Odontogenic myxoma is a rare benign neoplasm that originates from odontogenic ectomesenchyme. There is no standard of care and recurrences are frequent after conservative surgical procedures.

**Material and Methods:**

A retrospective study conducted at a single cancer center, with analysis of medical records of all patients diagnosed with odontogenic myxoma from 1980 to 2010, along with a literature review.

**Results:**

There were 14 patients with diagnosis of odontogenic myxoma (OM). Most patients were female (78.6%) and Caucasian (100%), with ages ranging from 7 to 51 years (21.6 ± 11.6 years). The time period between the first symptom and first consultation ranged from 0 to 60 months (19.4 ± 19.97 months). The most frequent complaints were increased local volume or failure to tooth eruption. The most common tumor site was the mandible (11 cases, 78.5%). About radiological findings, most lesions were multilocular (9 cases, 64.3%) and with imprecise limits (12 cases, 85.7%). Surgery was performed in all cases and curettage was the most applied technique (10 cases, 71.4%). Three patients underwent mandibulectomy and complex reconstructions including iliac crest microvascular flap. Three patients had postoperative complications and 4 had local recurrences of the tumor. The follow up time ranged from 12 to 216 months (112 ± 70.8 months). All patients are without clinical and radiographic evidence of disease.

**Conclusions:**

OM is a locally aggressive and rare tumor. There is no gold standard surgical management and the therapeutic decision should be individualized taking into account the characteristics and extension of the tumor.

** Key words:**Mandible, myxoma, odontogenic, odontogenic tumor.

## Introduction

Odontogenic myxoma (OM) is a rare benign tumor that originates from odontogenic ectomesenchyme (1,2). This disease affects mostly young adults between the second and third decades of life, but can occur at any age. It shows no predilection for sex (3-5).

The lesions can be found in the maxilla and mandible, the latter being most affected (1,5,6). Clinically, OM is characterized by presenting insidious and oligosymptomatic slow growth, but with locally aggressive behavior causing bone destruction, soft tissue infiltration and dental disorders (4,6). Diagnosis usually occurs inadvertently in occasional radiological examinations (3-5). OM is radiologically characterized by a radiolucent image having uni- or multilocular aspect and expansion of the cortical bone, associated with or without the presence of teeth with jagged or scalloped edges. It is important to carry out differential diagnosis including ameloblastoma, odontogenic keratocysts, aneurismal bone cyst and other dental-related diseases (4,5,7).

From a histological point of view, OM is as a tumor composed of round and spindle cells arranged amid loose myxoid stroma with few collagen fibers. In cases where there is a greater collagen component, it can be called fibromyxomas or myxofibromas (2,4,5).

Surgery is the main therapeutic modality of OM (8,9), ranging from less invasive procedures, such as curettage or marginal mandibulectomy up to extended resections. Reconstruction in minor defects is usually done with bone graft from the iliac crest or local mucosal flaps. In larger defects, reconstruction is usually done with microvascular fibula flaps (4,8,9).

The aim of this study is to analyze a series of 14 patients with odontogenic myxoma treated in a single institution.

Material and Methods

Fourteen patients with OM undergoing treatment at the A.C. Camargo Cancer Center, São Paulo, Brazil, from 1980 to 2010, were retrospectively reviewed. The reason for exclusion was the following: other diagnosis after pathological review and treatment not performed at the institution. The duration of signs and symptoms presented at diagnosis and comorbidities were also recorded. Lesions were evaluated clinically, and the findings related to the site, size, extension, image exams and macroscopic appearance were registered in the medical charts. We made descriptive statistical tests including mean, age ranges and standard deviation.

## Results

**Figure 1:**
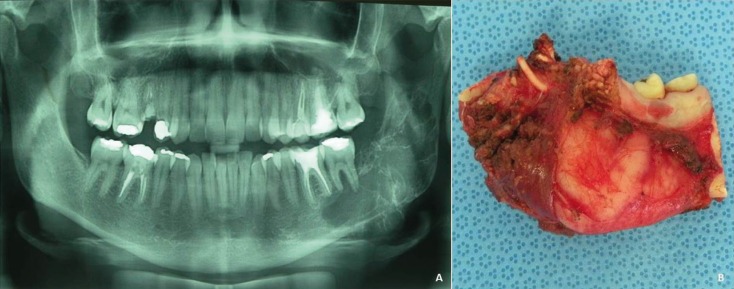
A) Panoramic radiograph of odontogenic myxoma affecting left mandibular molars, angle area and ascending ramus.External radicular resorption of the teeth by multinocular radiolucency. B) Specimen of odontogenic myxoma after surgical resection.

Most patients were female (11 cases, 78.6%) aged from 7 to 51 years (21.6 ± 11.6 years) and all were Caucasian. Among the 14 patients, the main complaints were local swelling in the mandible or maxilla, loss or absence of a dental element, with evolution time ranging from 0 to 60 months (19.4 ± 19.97 months). The most affected topography was the mandible (11 cases, 78.57%) with a predominance of the left side, anterior lateral (8 cases, 57.1%). Radiographically, lesions were radiolucent in aspect, being multilocular in 64.3% (Fig. 1A). The boundaries of the lesion were characterized as imprecise to imaging tests in 85.7% of cases and the size ranged from 2 to 6 cm (3.5 ± 1.3 cm). Clinicopathologic findings of all patients are shown in  Table 1.

**Table 1:**
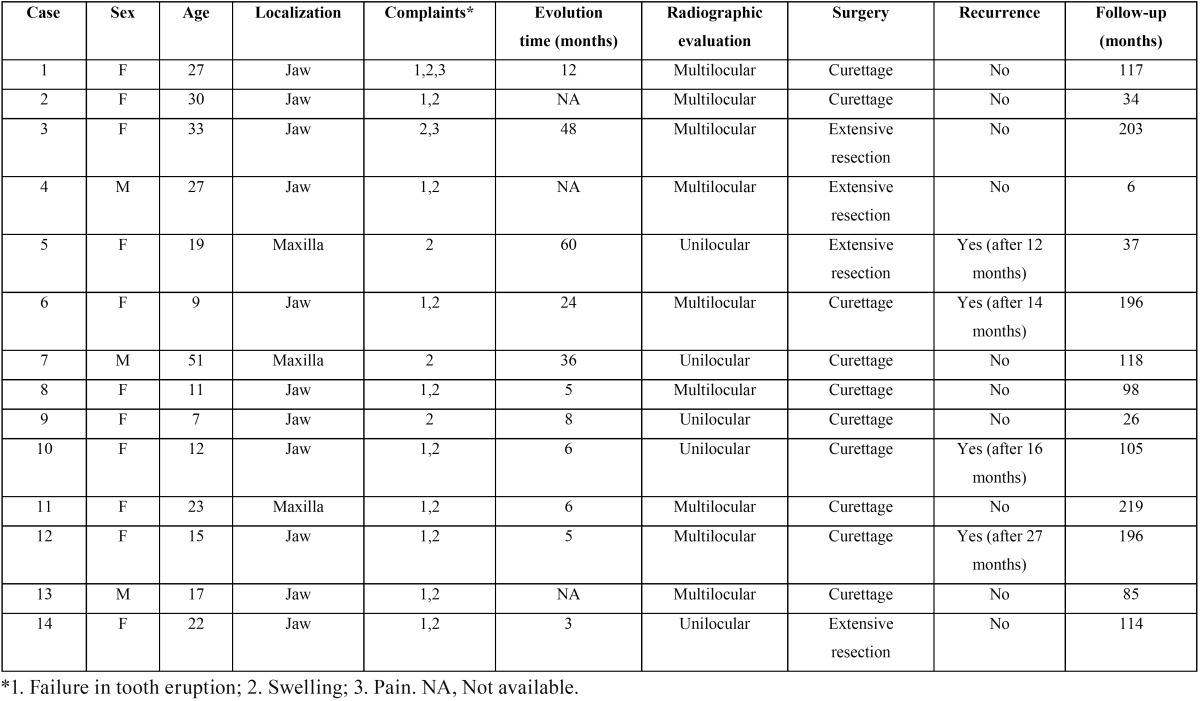
Clinicopathologic findings of all patients with odontogenic myxoma.

The surgical procedures were curettage (10 cases, 71.4%) or segmental resection (4 cases, 28.6%) (Fig. 1B). The four patients with segmental bone resection underwent reconstruction with microvascular flap of the iliac crest. The hospitalization period varied from 1 to 11 days (3.1 ± 3.0 days).

**Figure 2:**
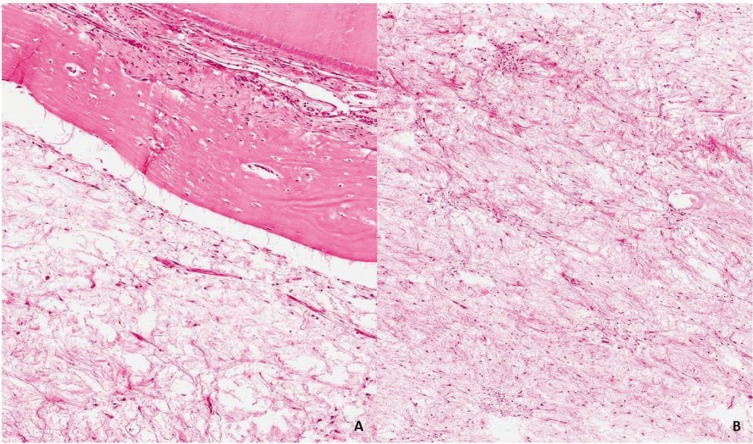
A) Odontogenic myxoma microscopically involving bone and dental structures (hematoxylin and eosin, original magnification ×100). B) Odontogenic myxoma showing proliferation of spindled and stellate cells in a mucoid-rich matrix (hematoxylin and eosin, original magnification ×200).

On macroscopic examination all tumors appeared whitish, soft or gelatinous in consistency. Microscopically, surgical specimens revealed a predominance of stellate and spindle cells in loose myxoid stroma with delicate fibrils and dense collagen fibers. There were rare mitotic figures or binucleated cells. Vascularity was minimal and no evidences of encapsulation in the present series (Fig. 2A-B).

Postoperative complications occurred in three patients (21.4%). The most common complication was transient dysfunction of the marginal mandibular branch of the facial nerve that occurred in two patients (14.2%). One of the patients underwent surgical resection which evolved postoperatively with loss of the microvascular flap.

During the follow-up period, ranging from 12 to 216 months (112 ± 70.8 months), four patients (28.4%) had disease recurrence (three were initially treated with curettage and one with resection). All patients with recurrent disease underwent salvage surgery with wide resection of the recurrence. In assessing the last follow up information, all patients were free of the disease.

## Discussion

OM represents less than 10% of all odontogenic tumors, being a locally invasive benign tumor, slow-growing, with well-defined pathologic characteristics and generally associated with tooth germ (5,8). In the series studied, it was found more frequently in young adults, ranging from 7 to 51 years (median, 19 years), and most were female, in agreement with the literature (4,5,8,10).

As stated in the reviewed literature, in our study mandible was also more frequently affected than the maxilla (11 and 3 cases respectively) (1,2,4,5,10). Among the most frequent complaints are increasing local volume, cortical bone drilling and invasion of soft tissues, mobility or absence of tooth and pain (5,7-10,). The radiographic pattern was of a multilocular lesion in 9 cases, 64.2%), described as multilocular radiolucent lesion with aspect of “soap bubbles” or “web spider”, reproducing the data from the literature (2,4,5,11).

The diagnosis must be confirmed by incisional biopsy to rule out intraosseous cystic lesions, central giant cell lesion, ameloblastoma, odontogenic keratocysts or other osteolytic lesions (2,6,8). Radiographic differential diagnosis must be done mainly with ameloblastoma, ameloblastic fibroma, odontogenic fibroma, central hemangioma or odontogenic keratocyst (11). The pathologic diagnosis consists of the presence of fusiform mesenchymal cells interspersed in a loose myxoid matrix and mucoid with surrounding residual bone (2,6,8,12,13).

Chondromyxoid fibroma and chondrosarcoma should be included in the microscopic differential diagnosis. Both lesions can have a myxoid stroma showing areas with chondrocytes residing in lacunae of cartilagenous material. Desmoplastic fibroma also should be considered in the differential diagnosis due to proliferation of spindle cells in a fibromyxoid stroma (11).

Treatment should be individualized, taking into account tumor location, size and presence of local invasion, preferring where possible, less invasive procedures initially as a marginal bone resection or curettage (4,5,12). Enucleation and isolated curettage can reach 25% recurrence rates (4). Studies, proposing better ways of rehabilitation with a lower psychosocial impact, advocate curettage with cryotherapy as an alternative to radical resection to minimize complications and not compromise treatment success (8,10). Radical surgery is indicated for more extensive lesions or locally invasive characteristics, besides being the approach of choice in recurrent lesions with recommended margins of 0.5-1 cm (4,10,14,15).

Ghosh *et al.* described a series of 10 cases with 50 years of follow-up, in which 9 patients underwent resection and 1 was treated conservatively with local excision, as this was the only case with recurrence (14).

In the present study we observed recurrences in cases that were treated either with curettage or bone resection, requiring additional salvage surgery with extended resection in 30% of patients who underwent curettage and in 25% of patients in whom resection was carried out as an initial procedure, corroborating literature data of the locally aggressive behavior of the tumor.

Reconstruction was done mainly with microvascular flap of the iliac crest, with loss of one to three flaps. Microsurgical fibula flap presents as an attractive and effective option for resections after extensive mandibular reconstructions (16).

OM is a locally aggressive and several factors, including gelatinous nature, absence of a capsule, tumor location and treatment approach may contribute to high recurrence rates. Hence, it has been recommended that patients should be followed closely for a long period.
